# Predictive value of baseline hematological and platelet indices for visual and anatomical outcomes in retinal vein occlusion: a retrospective analysis

**DOI:** 10.1186/s40942-026-00905-3

**Published:** 2026-07-31

**Authors:** Funda Yüksekyayla, Ali Şimşek, Ali Hakim Reyhan, Çağri Mutaf, Irfan Uzun, Evin Esin Kamışlı

**Affiliations:** 1 Department of Ophthalmology, Harran University Faculty of Medicine, Sanliurfa, Turkey; 2 Sanlıurfa Mehmet Akif İnan Training and Research Hospital, Sanliurfa, Türkiye

**Keywords:** Retinal vein occlusion, Central macular thickness (CMT), Mean platelet volüme (MPV), Plateletcrit (PCT), Intravitreal injections, Inflammatory biomarkers

## Abstract

**Purpose:**

To investigate the prognostic value of demographic characteristics, platelet activation markers, and systemic inflammatory indices in predicting anatomical and functional outcomes in patients with retinal vein occlusion (RVO) undergoing intravitreal therapy.

**Methods:**

The medical records of patients diagnosed with RVO and followed at the Department of Ophthalmology, Faculty of Medicine, Harran University, between January 2016 and January 2025 were retrospectively reviewed. After applying the inclusion and exclusion criteria, 65 eyes of 65 patients were included in the study. Patients were classified as having branch retinal vein occlusion (BRVO) or central retinal vein occlusion (CRVO) according to the site of venous occlusion. Baseline complete blood count parameters, platelet indices, including mean platelet volume (MPV), platelet distribution width (PDW), and plateletcrit (PCT), as well as inflammatory indices, including the neutrophil-to-lymphocyte ratio (NLR), platelet-to-lymphocyte ratio (PLR), and monocyte-to-lymphocyte ratio (MLR), were recorded. Anatomical outcomes were assessed by changes in central macular thickness (ΔCMT), whereas functional outcomes were evaluated by changes in best-corrected visual acuity (ΔlogMAR). Statistical analyses were performed using SPSS version 27.0.

**Results:**

The mean age of the patients was 62.65 ± 11.49 years, and 48 patients (73.8%) had BRVO. Mean central macular thickness decreased from 668.6 ± 224.3 μm at baseline to 456.4 ± 227.4 μm at the final follow-up. A significant positive correlation was observed between ΔCMT and ΔlogMAR (*r* = 0.443, *p* < 0.001).MPV showed a significant positive correlation with the early anatomical response, as reflected by the change in CMT between baseline and the first follow-up (*r* = 0.290, *p* = 0.019). PCT was significantly correlated with final visual outcomes (*r* = 0.313, *p* = 0.011). No significant associations were found between other hematological parameters or inflammatory indices and anatomical or functional outcomes. The mean age of patients in the CRVO group was significantly lower than that of patients in the BRVO group (*p* = 0.049).

**Conclusions:**

Platelet activation markers may be associated with early anatomical response and final visual outcomes in patients with RVO. In particular, MPV and PCT may serve as potential biomarkers for predicting treatment response. Because these parameters are readily available from routine complete blood count testing, they may provide clinically useful information for prognostic assessment and individualized treatment planning in patients with RVO. Further large-scale, prospective, multicenter studies are warranted to validate these findings.

## Introduction

Retinal vein occlusion (RVO) is the second most common retinal vascular disease causing visual loss worldwide after diabetic retinopathy [1, 2]. According to the anatomical site of involvement, RVO is classified into central retinal vein occlusion (CRVO) and branch retinal vein occlusion (BRVO). This clinical entity leads to significant visual morbidity through complications such as macular edema and retinal neovascularization [3].

Although the etiopathogenesis of RVO is multifactorial, the fundamental mechanism is widely accepted to be based on the components of Virchow’s triad, including venous stasis, hypercoagulability, and endothelial injury [1] In addition, it has been reported that at the level of the lamina cribrosa or arteriovenous crossing sites, mechanical compression of the vein by a rigid, atherosclerotic artery sharing a common adventitial sheath predisposes to turbulent blood flow and endothelial damage, thereby triggering thrombus formation [4].

Among the systemic risk factors predisposing to this pathological process, advanced age, hypertension, diabetes mellitus, and dyslipidemia—major atherosclerotic risk factors—play a critical role in the development of RVO and indicate an increased risk of systemic cardiovascular events in these patients [1]. In addition to factors such as obesity and smoking, oxidative stress and systemic inflammation have been shown to contribute to the pathogenesis by inducing vascular endothelial dysfunction [5, 6]. Particularly in younger patients, thrombophilic disorders, hyperhomocysteinemia, and genetic mutations in the coagulation cascade emerge as prominent etiological factors rather than classical cardiovascular risk factors [7].

It is increasingly recognized that the pathogenesis of RVO is a complex process in which systemic and local inflammation play an active role beyond the components of Virchow’s triad—venous stasis, endothelial injury, and hypercoagulability [2, 7]. Neutrophil-to-lymphocyte ratio (NLR), platelet-to-lymphocyte ratio (PLR), and monocyte-to-lymphocyte ratio (MLR), which can be easily derived from routine complete blood count parameters, are considered novel, low-cost biomarkers reflecting systemic inflammatory response, endothelial dysfunction, and physiological stress [6, 8] Although these indices have been associated with disease severity in atherosclerotic heart disease and various vascular occlusive conditions, MLR levels in particular have been shown to be an independent predictive factor for response to anti-VEGF therapy [8, 9].

Mean platelet volume (MPV), in particular, is an important indicator of platelet activation and production rate. Large-scale meta-analyses have confirmed that elevated MPV levels are significantly more pronounced in both CRVO and BRVO, and that larger platelets possess a higher prothrombotic potential [10].

Accordingly, the aim of the present study was to evaluate demographic and anthropometric parameters such as age, height, weight, and body mass index (BMI), as well as platelet activation markers (MPV) and systemic inflammatory indices (NLR, MLR, PLR) in patients diagnosed with RVO (BRVO and CRVO), and to investigate the impact and prognostic value of these parameters on anatomical (change in central macular thickness [CMT]) and functional (best-corrected visual acuity (VA) outcomes following intravitreal therapy [11].

## Material and methods

This retrospective study was conducted at the Department of Ophthalmology, Faculty of Medicine, Harran University, and was approved by the Harran University Non-Interventional Research Ethics Committee (Decision Date/No: 13.01.2025/01/42). The medical records of patients diagnosed with retinal vein occlusion (RVO) and followed up and treated between January 2016 and January 2025 were retrospectively reviewed. The study protocol was conducted in accordance with the principles of the Helsinki Declaration.

### Study population

The medical records of 250 consecutive patients diagnosed with retinal vein occlusion (RVO) between January 2016 and January 2025 were retrospectively reviewed. Patients were excluded if they met any of the following criteria: age under 18 years (*n* = 3), pregnancy (*n* = 2), severe anemia (*n* = 10), chronic inflammatory disease (*n* = 18), a history of blood transfusion within the previous 3 months (*n* = 4), or abnormal white blood cell and platelet parameters (*n* = 20). Based on previous literature and institutional laboratory reference ranges, abnormal hematological values were defined as a white blood cell count < 4.0 or > 11.0 × 10⁹/L and a platelet count < 150 or > 450 × 10⁹/L.

**Fig. 1:**
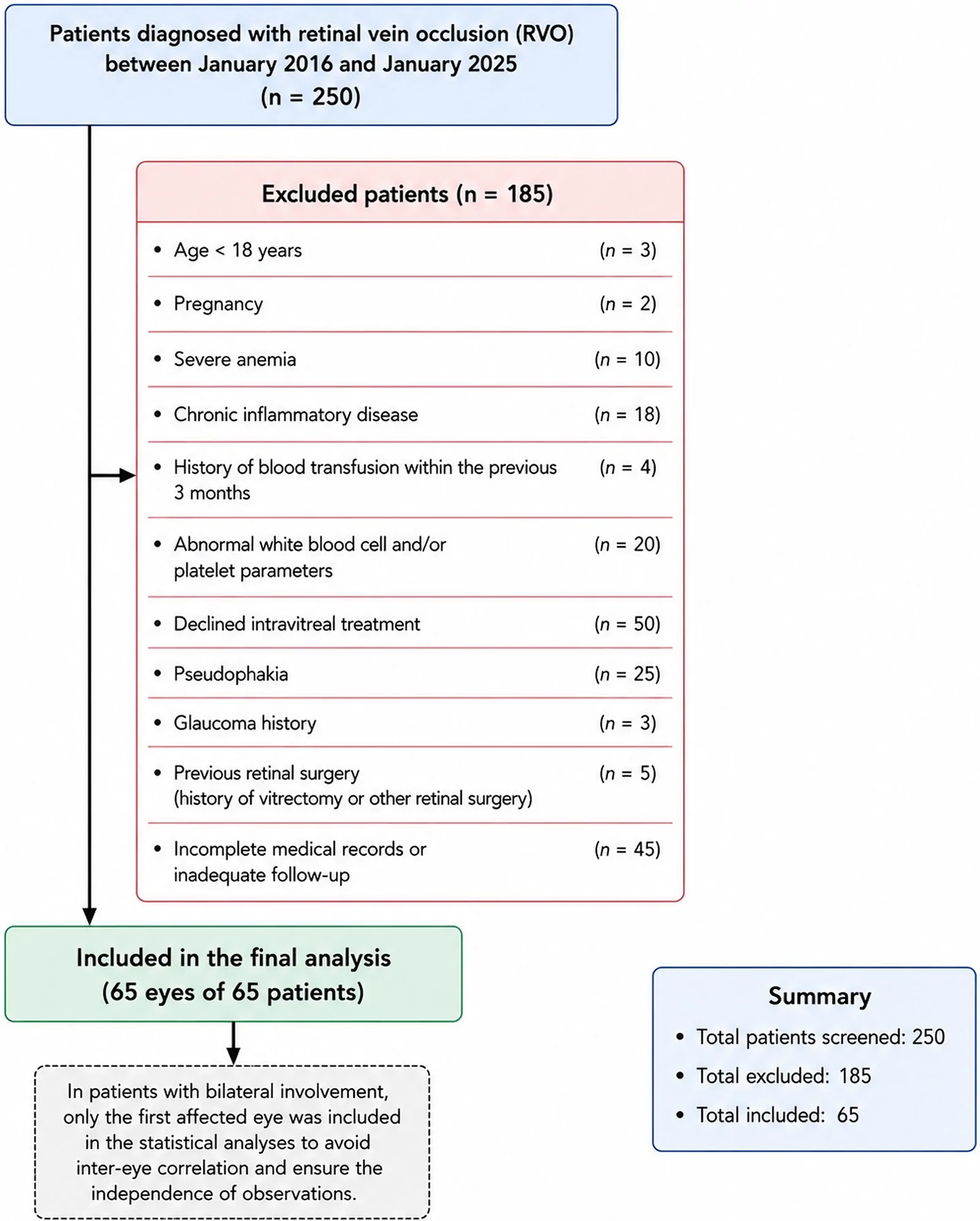
Flow diagram of patient selection. RVO: retinal vein occlusion

In addition, 50 patients who declined intravitreal treatment and 78 patients with incomplete medical records or insufficient follow-up data were excluded. Consequently, 65 eyes of 65 patients were included in the final analysis (Fig. 1).

**Fig. 2:**
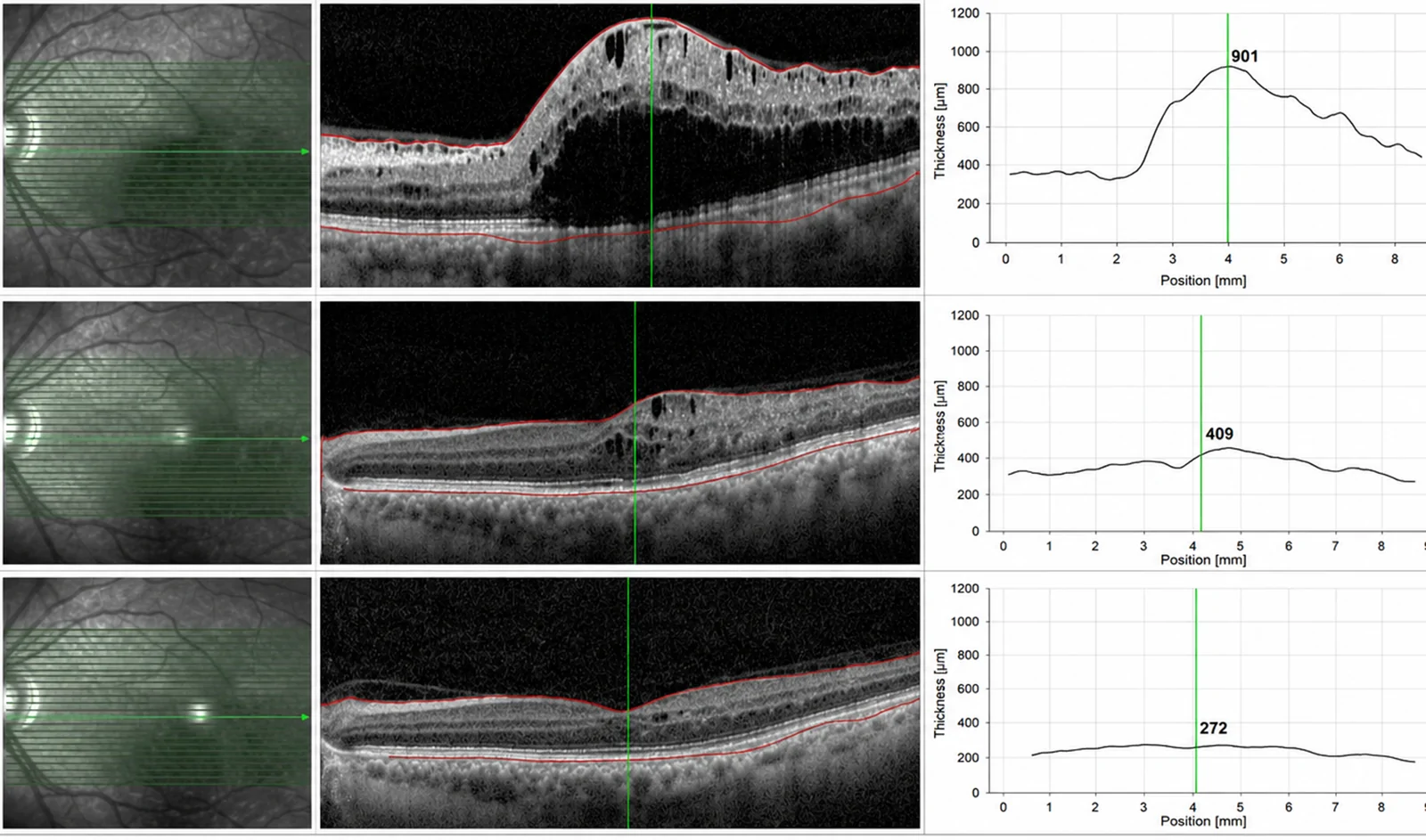
Optical coherencetomography findings demonstrating macular thickness change after IVI therapy in branchial retinal vein occlusion. (**A**) Baseline OCT scan showing increased central macular thickness (CMT) prior to treatment. (**B**) Final follow-up OCT scan obtained during the treatment course, demonstrating a marked reduction in central macular thickness. Images were obtained from the xxx Department Of OphtalmologyArchive’s

In patients with bilateral involvement, only the first affected eye was included in the statistical analyses to avoid inter-eye correlation and ensure the independence of observations. Patients were divided into two main groups based on the anatomical location of the vein occlusion: Branch Retinal Vein Occlusion (BRVO) and Central Retinal Vein Occlusion (CRVO) (Fig. 2).

#### Ophthalmological examination and imaging

All patients underwent a detailed ophthalmological examination at the time of diagnosis and during follow-up visits. Best corrected visual acuity at the initial examination (VA1), final visual acuity after treatment (finalVA), difference in visual acuity between the initial and final examinations (ΔVA) LogMAR (Logarithm of the Minimum Angle of Resolution) Intra ocular pressure (IOP) was measured using applanation tonometry. Central macular thickness (CMT) was measured using spectral-domain optical coherence tomography (SD-OCT) (Heidelberg Engineering, Heidelberg, Germany) at three predefined time points: baseline before treatment initiation (CMT1), after the first intravitreal treatment (CMT2), and at the final follow-up visit performed at least 6 months after treatment initiation (CMT3). The CMT3 value represented the central macular thickness measured at the patient’s last available examination during the follow-up period. Anatomical response was assessed by calculating the changes in CMT between these time points (ΔCMT). Fundus fluorescein angiography (FFA) was performed in all patients without contraindications to evaluate retinal perfusion status and to classify retinal vein occlusion as ischemic or non-ischemic. In patients with contraindications to fluorescein angiography, including a history of fluorescein allergy, severe renal impairment, or inability to undergo angiography, optical coherence tomography angiography (OCTA) was performed as an alternative imaging modality. The classification of ischemic and non-ischemic RVO was based on established criteria in the literature. Eyes with extensive capillary non-perfusion, defined as more than 10 disc areas of retinal non-perfusion in central retinal vein occlusion (CRVO) or more than 5 disc diameters of non-perfusion in branch retinal vein occlusion (BRVO) on FFA, were classified as ischemic. In cases evaluated by OCTA, eyes demonstrating marked enlargement of the foveal avascular zone and/or extensive areas of capillary dropout in the superficial and deep retinal capillary plexuses, consistent with previously published criteria, were classified as ischemic. Treatments were administered as needed (PRN). All patients were evaluated and managed by experienced retina specialists. Intravitreal treatment was administered on a pro re nata (PRN) basis according to the clinical findings and anatomical response.

Treatment was initiated with intravitreal bevacizumab (Avastin^®^, Genentech, South San Francisco, CA, USA). Following three consecutive monthly bevacizumab injections, patients with persistent or recurrent macular edema, inadequate anatomical improvement, or unsatisfactory visual response could undergo a treatment switch at the discretion of the treating retina specialist. In such cases, treatment was switched to either ranibizumab (Lucentis^®^, Novartis Pharma AG, Basel, Switzerland) or a dexamethasone intravitreal implant (Ozurdex^®^, Allergan, Irvine, CA, USA), according to the physician’s clinical judgment.

There after, all patients were followed using a PRN regimen, and additional intravitreal injections were administered in the presence of persistent or recurrent macular edema, increased central macular thickness on OCT, or deterioration in visual acuity attributable to macular edema.

Macular edema remission was defined as the absence of intraretinal and/or subretinal fluid on spectral-domain optical coherence tomography (SD-OCT), accompanied by a central macular thickness (CMT) of less than 300 μm or a stable dry macula without the need for additional treatment. Recurrence of macular edema was defined as the reappearance of intraretinal and/or subretinal fluid after a previously dry macula or an increase in CMT of ≥ 50 μm from the lowest recorded value, with or without an associated decrease in visual acuity, requiring additional treatment.

Laser photocoagulation (argon laser) was added in eyes with ischemic retinal findings or in cases in which macular edema persisted despite intravitreal treatment. The total number of intravitreal injections and the duration of follow-up for each patient were recorded.

### Laboratory analysis

Data were obtained from venous blood samples taken at the time of diagnosis and prior to treatment. Complete blood count (CBC) parameters, including white blood cell count (WBC), hemoglobin (HGB), platelet count (PLT), and platelet indices, including mean platelet volume (MPV), platelet distribution width (PDW), and plateletcrit (PCT), were obtained from EDTA-anticoagulated whole blood samples using a Sysmex XN-300 automated hematology analyzer (Sysmex Corporation, Kobe, Japan). The analyzer performs cell counting using the electrical impedance (Coulter principle) and optical laser light scatter methods. Platelet indices, including MPV, PDW, and PCT, were automatically calculated by the analyzer based on platelet volume measurements and reported according to the manufacturer’s specifications. Serum lipid profile high-density lipoprotein low-density lipoprotein (HDL, LDL), total cholesterol, triglycerides and coagulation tests such as prothrombin time (PT), activated partial thromboplastin time, (aPTT), International Normalized Ratio (INR) were analyzed using Standard biochemical methods.

### Statistical analysis

Statistical analysis of the data was performed using IBM SPSS Statistics 27.0 (Armonk, NY, USA). The normality of the variables was examined using the Kolmogorov-Smirnov and Shapiro-Wilk tests. In descriptive statistics, data showing a normal distribution were presented as mean ± Standard deviation (Mean ± SD), while data not showing a normal distribution were presented as median and minimum-maximum (Min-Max) values. Categorical data were expressed as number (n) and percentage (%). In the comparison of two independent groups (BRVO and CRVO), the Independent Samples t-Test was used for normally distributed data, and the Mann-Whitney U test was used for non-normally distributed data. The Chi-square test wasused in the comparison of categorical variables (gender, comorbidities, type of treatment). The direction and strength of the association between the variables were assessed using Pearson or Spearman correlation analyses. In all analyses, *p* < 0.05 was considered statistically significant.

## Results

### Baseline characteristics

A total of 65 eyes of 65 patients with retinal vein occlusion (RVO) were included in the study. Of these, 48 patients (73.8%) had branch retinal vein occlusion (BRVO) and 17 (26.2%) had central retinal vein occlusion (CRVO). The mean age was 62.65 ± 11.49 years. Baseline central macular thickness (CMT1) was 663.98 ± 229.96 μm. Baseline demographic, clinical, and hematological characteristics are summarized in Table 1.

**Table 1:** Baseline demographic, clinical, hematological, coagulation, biochemical, and treatment characteristics according to retinal vein occlusion subtype

Variable	Overall (*n* = 65)	BRVO (*n* = 48)	CRVO (*n* = 17)	*P* value
**Demographic characteristics**				
Age (years)	62.65 ± 11.49	64.98 ± 9.00	56.06 ± 15.09	**0.032**
Female, n (%)	32 (49.2)	23(47.9)	9(52.9)	0.73
Male, n (%)	33(50.8)	25(52.1)	8(47.1)	
Right eye, n (%)	32 (49.2)	25 (52.1)	7 (41.2)	0.7
Left eye, n (%)	30 (46.2)	21 (43.8)	9(52.9)	
Bilateral	3(4.6)	2(4.2)	1(5.9)	
BMI (kg/m²)	27.78 ± 2.48	27.97 ± 2.72	27.22 ± 1.57	0.173
**Clinical characteristics**				
Baseline BCVA (logMAR)	1.26 ± 0.75	1.14 ± 0.69	1.60 ± 0.83	**0.049**
Intraocular pressure (mmHg)	16.26 ± 3.52	16.50 ± 3.78	15.59 ± 2.65	0.363
Symptom duration (days)	26.26 ± 47.82	27.29 ± 49.69	23.35 ± 43.39	0.491
Baseline CMT (µm)	668.60 ± 224.30	643.65 ± 206.23	739.06 ± 263.00	0.133
Month-1 CMT (µm)	390.09 ± 202.48	400.81 ± 214.04	359.82 ± 167.66	0.633
Final CMT (µm)	456.40 ± 227.37	436.46 ± 215.72	512.71 ± 255.97	0.238
ΔCMT (Baseline–Month 1) (µm)	−278.51 ± 237.45	−242.83 ± 228.80	−379.24 ± 255.59	**0.045**
ΔCMT (Baseline–Final) (µm)	−212.20 ± 186.88	−207.19 ± 191.33	−226.35 ± 176.34	0.719
ΔCMT (Month 1–Final) (µm)	66.31 ± 206.86	35.65 ± 193.24	152.88 ± 225.07	**0.044**
**Hematological parameters**				
Platelet count (×10³/µL)	283.34 ± 97.06	277.21 ± 88.27	300.65 ± 119.84	0.396
MPV (fL)	8.90 ± 2.28	9.19 ± 1.97	8.08 ± 2.89	0.086
PCT (%)	0.34 ± 0.78	0.24 ± 0.08	0.61 ± 1.53	0.754
PDW	49.26 ± 264.41	60.38 ± 307.75	17.84 ± 3.53	0.573
NLR	13.05 ± 55.09	15.93 ± 63.45	4.41 ± 5.36	0.914
PLR	155.81 ± 171.97	144.44 ± 163.53	187.91 ± 195.59	0.817
MLR	8.34 ± 48.13	7.56 ± 50.48	10.54 ± 42.12	0.339
**Coagulation parameters**				
INR	0.98 ± 0.09	0.97 ± 0.08	1.00 ± 0.11	0.175
aPTT (s)	23.73 ± 2.91	23.95 ± 2.82	23.09 ± 3.14	0.296
**Treatment characteristics**				
Number of intravitreal injections	4.25 ± 5.21	4.19 ± 5.19	4.41 ± 5.44	0.799
Follow-up duration (months)	32.32 ± 38.68	30.04 ± 37.73	38.76 ± 41.75	0.429

*Data are presented as mean ± standard deviation or number (%). BCVA: best-corrected visual acuity; BMI: body mass index; BRVO: branch retinal vein occlusion; CRVO: central retinal vein occlusion; CMT: central macular thickness; MPV: mean platelet volume; PCT: plateletcrit; PDW: platelet distribution width; NLR: neutrophil-to-lymphocyte ratio; PLR: platelet-to-lymphocyte ratio; MLR: monocyte-to-lymphocyte ratio; INR: international normalized ratio; aPTT: activated partial thromboplastin time; IVI: intravitreal injection. Independent samples t-test or Mann–Whitney U test was used for continuous variables, and χ² or Fisher’s exact test for categorical variables. Statistical significance was defined as *P* < 0.05

Comparison between RVO subtypes demonstrated that patients with CRVO were younger than those with BRVO (56.06 ± 15.09 vs. 64.98 ± 9.00 years, *p* = 0.032). Baseline visual acuity was also significantly worse in the CRVO group in the unadjusted analysis. However, after false discovery rate (FDR) correction for multiple comparisons, no significant differences remained between the two groups.

### Treatment characteristics

All patients received intravitreal treatment using a pro re nata (PRN) regimen following three consecutive loading doses of bevacizumab. Treatment response was evaluated by an experienced retina specialist, and switching to another anti-VEGF agent or a dexamethasone implant was performed at the physician’s discretion in eyes showing inadequate anatomical or functional response. Laser photocoagulation was applied when indicated in eyes with ischemic retinal changes or persistent macular edema.

The mean number of intravitreal injections and the follow-up duration are presented in Table 1. No significant differences in treatment characteristics were observed between the BRVO and CRVO groups.

### Anatomical outcomes

Mean central macular thickness decreased from 663.98 ± 229.96 μm at baseline (CMT1) to 387.03 ± 206.33 μm after the first intravitreal treatment (CMT2), with a final CMT (CMT3) of 464.52 ± 232.32 μm at the last follow-up visit.

**Fig. 3:**
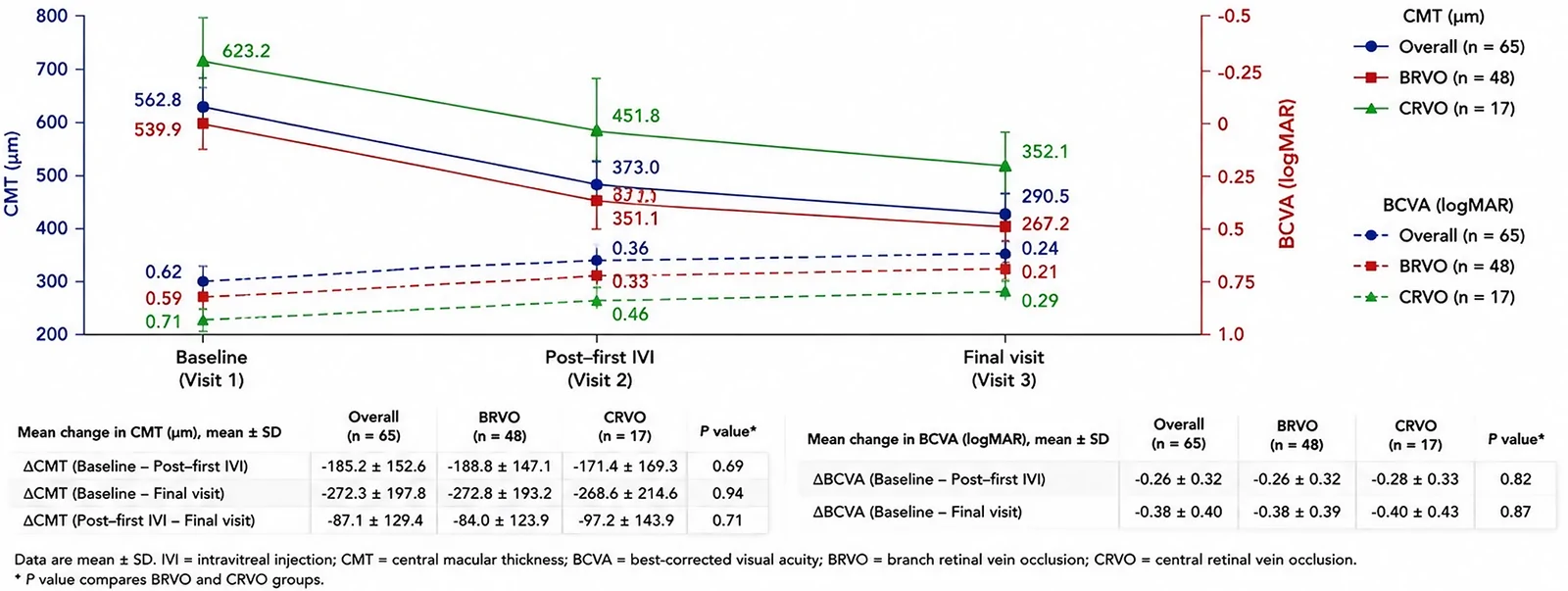
Changes in CMT and BCVA after treatment

Correlation analysis demonstrated a significant association between anatomical improvement and functional improvement, with greater reductions in CMT being associated with larger improvements in visual acuity (*r* = 0.443, *p* < 0.001) (Fig. 3).

Among platelet indices, MPV demonstrated a weak inverse correlation with early anatomical response (ΔCMT12) in the unadjusted Pearson analysis (*r* = − 0.313, *p* = 0.011). However, this association was no longer significant after FDR and Bonferroni correction for multiple testing. Likewise, PCT showed only a borderline association with ΔCMT, whereas PDW and platelet count were not significantly associated with anatomical outcomes.

**Fig. 4:**
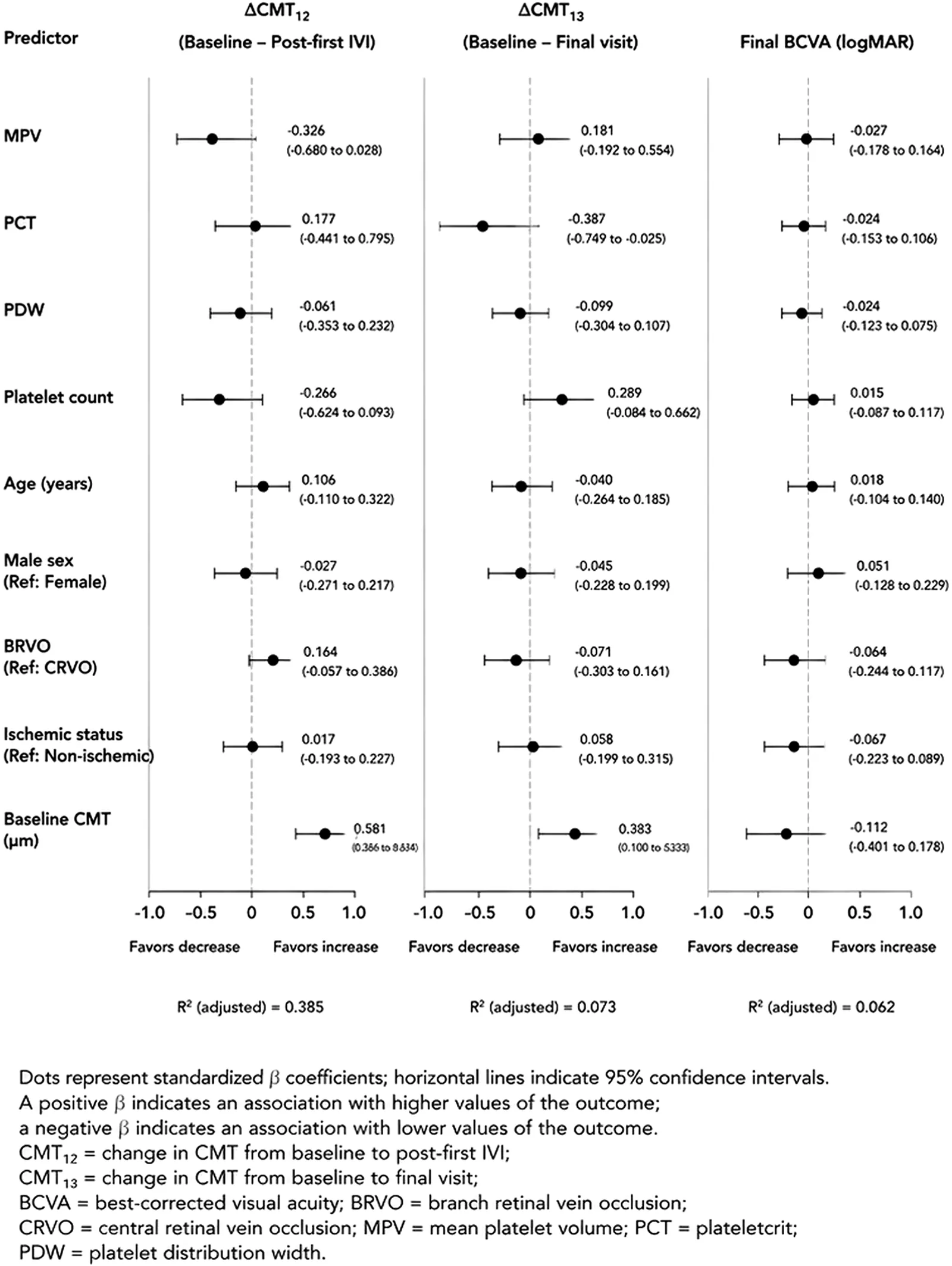
Multivariable linear regression analyses outcomes **(s**tandardized β with 95% CI)

In multivariable linear regression analysis, baseline CMT was the only independent predictor of both early and final anatomical outcomes (Fig. 4). None of the platelet indices, including MPV, PCT, PDW, or platelet count, independently predicted anatomical response after adjustment for age, sex, RVO subtype, ischemic status, and baseline CMT (Table 2).

**Table 2:** Multivariable linear regression analysis for anatomical outcomes

**Outcome**	**Predictor**	**>β**	**>B (95% CI)**	***P*** ** value**
CMT after first IVI (CMT2)	MPV	0.388	38.92 (− 16.51 to 94.34)	0.165
	PCT	−0.210	−500.57 (− 2279.11 to 1277.97)	0.575
	PDW	0.072	0.06 (− 0.13 to 0.24)	0.551
	Platelet count	0.316	0.66 (− 1.14 to 2.47)	0.466
	Age	−0.127	−2.24 (− 6.82 to 2.34)	0.331
	Male sex	0.032	12.89 (− 89.91 to 115.70)	0.802
	BRVO	−0.195	−89.25 (− 209.04 to 30.53)	0.141
	Ischemic status	−0.021	−8.56 (− 112.89 to 95.77)	0.870
	**Baseline CMT**	**0.441**	**0.390 (0.166 to 0.614)**	**0.001**
Adjusted R² = 0.128				
Final CMT (CMT3)	MPV	−0.149	−17.15 (− 70.59 to 36.29)	0.523
	PCT	0.319	865.99 (− 850.92 to 2582.91)	0.316
	PDW	0.082	0.07 (− 0.11 to 0.25)	0.432
	Platelet count	−0.238	−0.56 (− 2.30 to 1.18)	0.521
	Age	0.033	0.66 (− 3.75 to 5.07)	0.765
	Male sex	0.037	16.73 (− 82.84 to 116.29)	0.738
	BRVO	0.058	30.08 (− 85.27 to 145.43)	0.603
	Ischemic status	−0.048	−22.42 (− 123.58 to 78.74)	0.659
	**Baseline CMT**	**0.680**	**0.683 (0.467 to 0.900)**	**< 0.001**
Adjusted R² = 0.371				
ΔCMT12	MPV	−0.326	−38.92 (− 94.34 to 16.51)	0.165
	PCT	0.177	500.57 (− 1277.97 to 2279.11)	0.575
	PDW	−0.061	−0.06 (− 0.24 to 0.13)	0.551
	Platelet count	−0.266	−0.66 (− 2.47 to 1.14)	0.466
	Age	0.106	2.24 (− 2.34 to 6.82)	0.331
	Male sex	−0.027	−12.89 (− 115.70 to 89.91)	0.802
	BRVO	0.164	89.25 (− 30.53 to 209.04)	0.141
	Ischemic status	0.017	8.56 (− 95.77 to 112.89)	0.870
	**Baseline CMT**	**0.581**	**0.610 (0.386 to 0.834)**	**< 0.001**
Adjusted R² = 0.385				
ΔCMT13	MPV	0.181	17.15 (− 36.29 to 70.59)	0.523
	PCT	−0.387	−865.99 (− 2582.91 to 850.92)	0.316
	PDW	−0.099	−0.07 (− 0.25 to 0.11)	0.432
	Platelet count	0.289	0.56 (− 1.18 to 2.30)	0.521
	Age	−0.040	−0.66 (− 5.07 to 3.75)	0.765
	Male sex	−0.045	−16.73 (− 116.29 to 82.84)	0.738
	BRVO	−0.071	−30.08 (− 145.43 to 85.27)	0.603
	Ischemic status	0.058	22.42 (− 78.74 to 123.58)	0.659
	**Baseline CMT**	**0.383**	**0.317 (0.100 to 0.533)**	**0.005**
Adjusted R² = 0.073				
**Model performance**				
**Outcome**	**Adjusted R²**	**Model significance**		
CMT2	0.128	*p* = 0.051		
CMT3	0.371	*p* < 0.001		
ΔCMT12	0.385	*p* < 0.001		
ΔCMT13	0.073	*p* = 0.155		

Models were adjusted for age, sex, RVO subtype, ischemic status, baseline CMT, MPV, PDW, PCT, and platelet count. Data are presented as B (95% CI), standardized β, and P values. ΔCMT12 and ΔCMT13 represent changes in CMT from baseline to the first and final follow-up visits, respectively. Significant results are shown in bold

### Functional outcomes

Best-corrected visual acuity improved during follow-up. Nevertheless, no significant correlations were observed between platelet indices and baseline visual acuity, final visual acuity, or changes in visual acuity (ΔlogMAR). Similarly, multivariable regression analysis demonstrated that none of the evaluated hematological parameters independently predicted functional improvement (Table 3).

**Table 3:** Multivariable linear regression analysis for functional outcome (ΔlogMAR)

**Predictor**	**B (95% CI)**	**Standardized β**	***P*** ** value**	**FDR-adjusted P**
MPV	−0.007 (− 0.178 to 0.164)	−0.027	0.932	0.976
PCT	−0.153 (− 5.635 to 5.329)	−0.024	0.956	0.976
PDW	−0.000 (− 0.001 to 0.001)	−0.024	0.860	0.955
Platelet count	0.000 (− 0.005 to 0.006)	0.015	0.976	0.976
Age	0.001 (− 0.013 to 0.015)	0.018	0.904	0.969
Sex	0.056 (− 0.261 to 0.373)	0.051	0.725	0.950
RVO subtype	−0.079 (− 0.448 to 0.290)	−0.064	0.670	0.950
Ischemic status	−0.076 (− 0.397 to 0.246)	−0.067	0.638	0.950
Baseline CMT	−0.000 (− 0.001 to 0.000)	−0.112	0.442	0.950
**Model summary**				
**n**	**R²**	**Adjusted R²**	**Model P value**	
65	0.027	−0.132	0.996	

The dependent variable was the change in best-corrected visual acuity (ΔlogMAR) between baseline and the final follow-up. B represents the unstandardized regression coefficient with the corresponding 95% confidence interval (CI), whereas β denotes the standardized regression coefficient. P values were adjusted using the Benjamini–Hochberg false discovery rate (FDR)

### Hematologic correlations

**Fig. 5:**
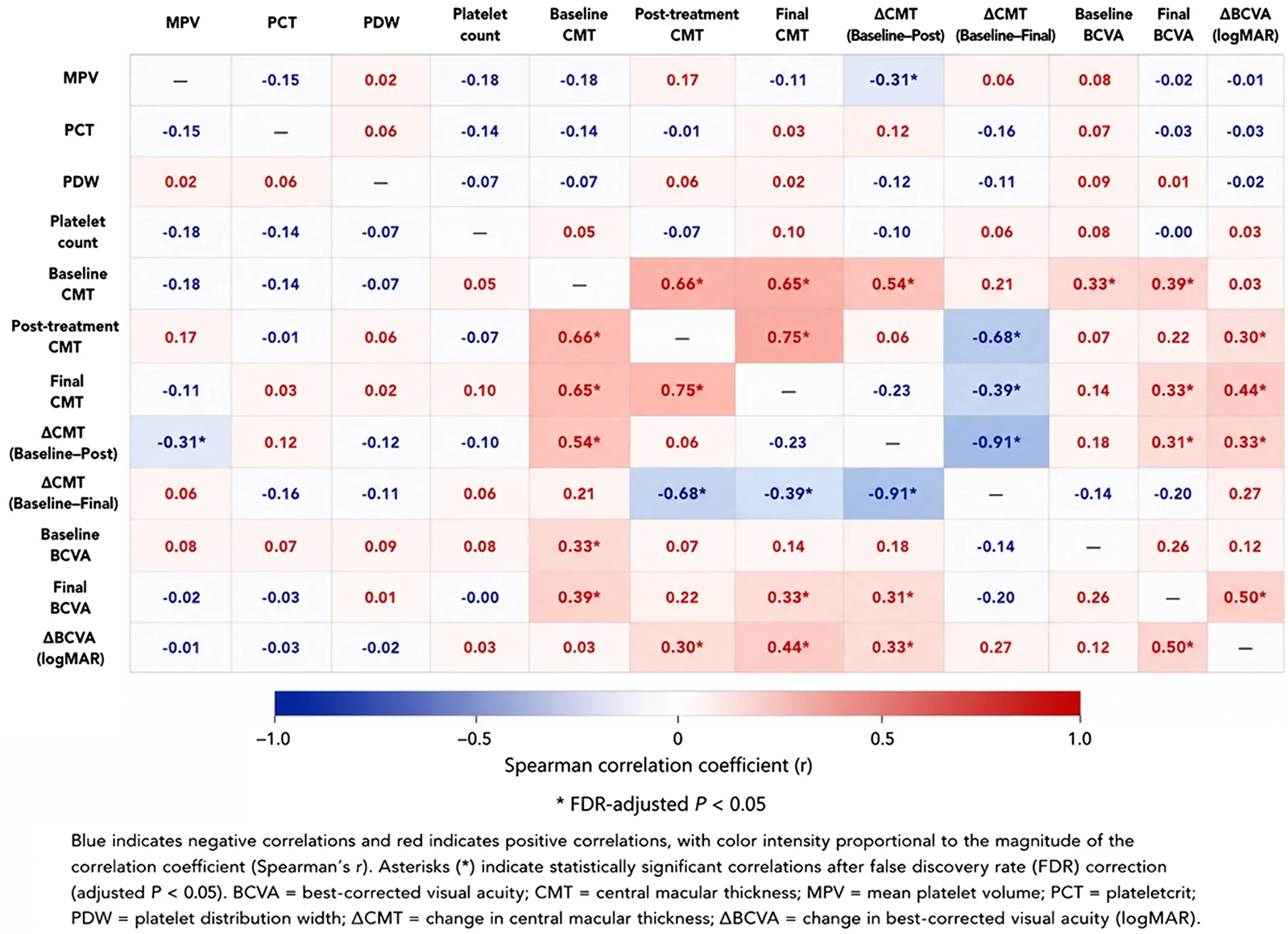
Spearman correlation heatmap showing the relationships between platelet indices, anatomical outcomes (CMT), and functional outcomes (BCVA) in patients with retinal vein occlusion

Correlation analyses evaluating hematological and platelet-related biomarkers are summarized in. MPV, PCT, PDW, platelet count, NLR, PLR, and MLR were analyzed in relation to anatomical and functional outcomes. Although MPV demonstrated a significant correlation with early anatomical response in the unadjusted analysis, this association did not remain significant after correction for multiple comparisons. Likewise, no significant correlations were identified between inflammatory indices (NLR, PLR, and MLR) and either anatomical or functional outcomes (Fig. 5).

Overall, after adjustment for multiple testing and potential confounding variables, baseline CMT remained the only consistent predictor of anatomical outcome, whereas none of the evaluated hematological biomarkers independently predicted treatment response (Figs. 4 and 5).

## Discussion

The present study evaluated whether routinely available hematological biomarkers could predict anatomical and functional responses to intravitreal therapy in retinal vein occlusion (RVO). The principal finding was that none of the evaluated platelet indices (MPV, PDW, PCT, and platelet count), coagulation parameters, or inflammatory ratios independently predicted treatment response after adjustment for clinical confounders and correction for multiple comparisons. Instead, baseline central macular thickness (CMT) emerged as the only independent predictor of anatomical outcome. Nevertheless, correlation analyses demonstrated that higher MPV was associated with a less favorable early anatomical response, whereas PCT was associated with final visual acuity, suggesting that platelet activation may still reflect disease severity despite its limited independent prognostic value.

Intravitreal therapy resulted in a mean **30.3% reduction in CMT** together with a **0.44 logMAR improvement** in visual acuity, confirming the effectiveness of current treatment strategies for RVO-associated macular edema. These findings are consistent with previous studies demonstrating that anatomical improvement is closely associated with functional recovery, although the relationship is influenced by retinal structural integrity in addition to retinal thickness [12, 13]. Likewise, the magnitude of CMT reduction observed in our cohort is comparable with previous meta-analyses and real-world studies reporting anatomical improvements of approximately 30–40% following anti-VEGF therapy [14–16]. In our cohort, bevacizumab was the most frequently administered anti-VEGF agent (63.1%), and the anatomical outcomes were similar to those reported in routine clinical practice. Previous real-world studies have also shown that most patients require approximately six to eight intravitreal injections during the first treatment year, with injection frequency gradually decreasing thereafter [17, 18].

Systemic vascular risk factors are well-established contributors to RVO development. Previous clinical studies have consistently identified hypertension, diabetes mellitus, dyslipidemia, thrombophilia, and aging as major determinants of disease occurrence and progression [3, 7, 8, 19]. More recently, attention has shifted toward platelet activation as an additional mechanism contributing to retinal vascular occlusion.

**Pinna et al.**, demonstrated that larger platelets are metabolically and enzymatically more active, contain more dense granules, and produce higher amounts of thromboxane A₂ and serotonin, thereby promoting thrombus formation [6]. Experimental studies have further shown that activated platelets express increased glycoprotein Ib and IIb/IIIa receptors, resulting in enhanced platelet aggregation and endothelial dysfunction [20, 21].

Previous studies investigating platelet indices in RVO have produced conflicting findings. Although elevated MPV has been consistently reported in patients with RVO compared with healthy controls the large meta-analysis by **Liu et al.**, concluded that platelet indices have only modest diagnostic performance and should not be used as standalone biomarkers [6, 22]. Our findings complement these observations by showing that higher MPV values were associated with a poorer early anatomical response; however, this association disappeared after multivariable adjustment, suggesting that MPV reflects disease severity rather than independently determining treatment response.

The association between elevated MPV and reduced early resolution of macular edema may be explained by persistent platelet activation and sustained microvascular occlusion. Hyperactive platelets release prothrombotic mediators that may perpetuate endothelial dysfunction and blood-retinal barrier disruption. Furthermore, inflammatory cytokines, including IL-6 and IL-8, have been shown to contribute substantially to persistent vascular permeability in RVO-associated macular edema [2]. Accordingly, elevated MPV may represent not only a marker of thrombosis but also an indicator of ongoing thrombo-inflammatory activity.

Interestingly, patients with CRVO in our cohort were significantly younger than those with BRVO. This observation is supported by cardiovascular literature demonstrating that elevated MPV predicts thrombotic events even in younger individuals without classical cardiovascular risk factors [23]. Together with the findings of **Liu et al.**, our results suggest that platelet activation may play a proportionally greater role in younger patients with CRVO than age-related vascular degeneration alone [22].

The inverse correlation observed between platelet count and MPV is compatible with accelerated platelet turnover secondary to peripheral platelet consumption. Elevated MPV has been shown to correlate with increased immature platelet fraction (IPF), which consists of newly released platelets with greater thrombotic potential [24, 25]. Because IPF was not evaluated in the present study, future prospective investigations incorporating this parameter may provide additional insights into platelet activation in RVO.

A negative correlation was also observed between early anatomical improvement and aPTT values. Shortened coagulation times may reflect increased thrombin generation and a hypercoagulable state [9]. Experimental evidence further suggests that coagulation activation contributes to endothelial dysfunction and persistent vascular permeability [26]. Although this association requires confirmation, these findings support a potential contribution of coagulation activity to treatment resistance.

Regarding functional outcomes, plateletcrit (PCT) was significantly associated with final visual acuity, whereas no significant relationship was observed between hematological parameters and visual acuity improvement. Because PCT reflects the total circulating platelet mass, it may indirectly represent the thrombotic burden at disease onset ([27]. Nevertheless, visual recovery is influenced by multiple additional factors, including retinal ischemia, photoreceptor integrity, and irreversible structural damage. Consequently, platelet-derived biomarkers appear to reflect disease severity more accurately than treatment-related visual improvement, consistent with previous observations [22].

The mean body mass index of our cohort was **27**,**78 kg/m²**, indicating that the study population was generally overweight. Obesity is known to promote chronic low-grade inflammation, endothelial dysfunction, and platelet activation, thereby creating a prothrombotic environment [9, 24]. Although BMI was not independently associated with treatment outcomes in our analyses, excess body weight may represent an additional metabolic factor contributing to platelet activation in patients with RVO.

The strengths of this study include the simultaneous evaluation of multiple platelet-derived, inflammatory, and coagulation biomarkers together with comprehensive multivariable analyses adjusted for major clinical confounders. Unlike many previous studies that primarily investigated disease susceptibility, our study focused specifically on predictors of anatomical and functional treatment response.

### Limitations

When interpreting the results of this study, several limitations should be considered. First, this was a retrospective, single-center study with a relatively small sample size, particularly in the CRVO subgroup, which may have limited the statistical power of subgroup analyses. Second, platelet indices were evaluated only at baseline using a single complete blood count obtained at diagnosis; serial measurements during follow-up were not available. Third, although multivariable regression analyses and false discovery rate (FDR) correction were performed to reduce potential confounding and type I error, residual confounding inherent to retrospective observational studies cannot be completely excluded. In addition, information regarding certain factors that may influence platelet indices, including antiplatelet medication use, chronic inflammatory diseases, and other unmeasured systemic conditions, was not consistently available. Finally, treatment strategies reflected real-world clinical practice and included different intravitreal agents according to physician discretion, and follow-up duration varied among patients. Therefore, the present findings should be considered hypothesis-generating and require validation in larger prospective multicenter studies with standardized treatment protocols and longitudinal assessment of platelet indices.

When interpreting the results of this study, several limitations should be considered. First, the retrospective, single-center design and the relatively small sample size limited the number of patients available for subgroup analyses. Second, platelet indices were evaluated indirectly, and only baseline hemogram values obtained at the time of diagnosis were analyzed; serial measurements during and after treatment were not available. In addition, although metabolic parameters such as lipid levels and body mass index (BMI) were recorded, certain environmental factors that may influence platelet function—such as diet, life style habits, or the use of over-the-counter medications—could not be fully standardized due to the retrospective nature of thestudy. These factors should be taken into account when interpreting the findings.

## Conclusion

In conclusion, although platelet activation and systemic inflammation are recognized contributors to the pathophysiology of retinal vein occlusion (RVO), routinely available hematological biomarkers—including MPV, PDW, PCT, platelet count, NLR, PLR, and MLR—did not independently predict anatomical or functional outcomes following intravitreal therapy in our cohort after adjustment for potential confounders. Baseline central macular thickness remained the only independent predictor of anatomical response, suggesting that retinal structural characteristics provide greater prognostic value than systemic hematological indices. Nevertheless, the observed trends in platelet-related biomarkers warrant further investigation. Large-scale prospective multicenter studies incorporating serial hematological measurements and more specific markers of platelet activation, such as the immature platelet fraction (IPF), together with retinal imaging biomarkers and molecular inflammatory profiling, are needed to better define the potential role of systemic biomarkers in predicting treatment response and personalizing management strategies for patients with RVO.

## Data Availability

Data supporting the findings of this study are available from the corresponding author upon reasonable request.

## Declarations

### AI statement

AI-assisted tools were used only for English language and grammar correction during manuscript preparation and were not involved in study design, data collection, data analysis, or manuscript generation.

## Abbreviations

aPTT: Activated Partial Thromboplastin Time
BCVA: Best-Corrected Visual Acuity
BMI: Body Mass Index
BRVO: Branch Retinal Vein Occlusion
CBC: Complete Blood Count
CI: Confidence Interval
CMT: Central Macular Thickness
CRVO: Central Retinal Vein Occlusion
EDTA: Ethylenediaminetetraacetic Acid
FDR: False Discovery Rate
FFA: Fundus Fluorescein Angiography
HGB: Hemoglobin
HDL: High-Density Lipoprotein
IL: Interleukin
INR: International Normalized Ratio
IOP: Intraocular Pressure
IPF: Immature Platelet Fraction
IVI: Intravitreal Injection
LDL: Low-Density Lipoprotein
LogMAR: Logarithm of the Minimum Angle of Resolution
MLR: Monocyte-to-Lymphocyte Ratio
MPV: Mean Platelet Volume
NLR: Neutrophil-to-Lymphocyte Ratio
OCT: Optical Coherence Tomography
OCTA: Optical Coherence Tomography Angiography
PCT: Plateletcrit
PDW: Platelet Distribution Width
PLR: Platelet-to-Lymphocyte Ratio
PRN: Pro Re Nata
PT: Prothrombin Time
RVO: Retinal Vein Occlusion
SD: Standard Deviation
SD-OCT: Spectral-Domain Optical Coherence Tomography
VA: Visual Acuity
VEGF: Vascular Endothelial Growth Factor
WBC: White Blood Cell Count
